# Hemoglobin, Body Mass Index, and Age as Risk Factors for Paclitaxel- and Oxaliplatin-Induced Peripheral Neuropathy

**DOI:** 10.1001/jamanetworkopen.2020.36695

**Published:** 2021-02-15

**Authors:** David Mizrahi, Susanna B. Park, Tiffany Li, Hannah C. Timmins, Terry Trinh, Kimberley Au, Eva Battaglini, David Wyld, Robert D. Henderson, Peter Grimison, Helen Ke, Peter Geelan-Small, Julie Marker, Brian Wall, David Goldstein

**Affiliations:** 1 Prince of Wales Clinical School, UNSW Medicine, University of New South Wales Sydney, Sydney, Australia; 2 Brain and Mind Centre, University of Sydney, Sydney, Australia; 3 Royal Brisbane and Women’s Hospital, Brisbane, Australia; 4 Faculty of Medicine, University of Queensland, Brisbane, Australia; 5 Chris O’Brien Lifehouse, Sydney, Australia; 6 Sydney Medical School, University of Sydney, Sydney, Australia; 7 Mark Wainwright Analytical Centre, University of New South Wales, Kensington, Australia; 8 The Australasian Gastro-Intestinal Trials Group Consumer Advisory Panel, Sydney, Australia; 9 Department of Medical Oncology, Prince of Wales Hospital, Sydney, Australia

## Abstract

**Importance:**

Chemotherapy-induced peripheral neuropathy (CIPN) is a debilitating adverse effect of neurotoxic cancer treatments including taxanes and platinum agents. Limited knowledge exists of potential prechemotherapy factors associated with CIPN development.

**Objective:**

To identify the association of pretreatment blood-based and clinical factors with CIPN persistence in patients who received paclitaxel or oxaliplatin.

**Design, Setting, and Participants:**

This cohort study assessed pretreatment blood-based clinical factors and demographic characteristics of 333 patients treated with paclitaxel and oxaliplatin chemotherapy at urban multicenter cancer clinics and academic institutions in Australia between September 2015 and February 2020. Comprehensive neuropathy assessments were undertaken 3 to 12 months posttreatment. Posttreatment CIPN severity was compared with blood-based factors within 30 days prior to commencing chemotherapy. Data were analyzed between March and December 2020.

**Exposures:**

Paclitaxel or oxaliplatin chemotherapy.

**Main Outcomes and Measures:**

CIPN was measured using composite neurological grading scales, nerve conduction studies, and assessments of fine motor skills (grooved pegboard test), sensory function (grating orientation test and 2-point discrimination), and patient-reported outcomes. Independent samples *t* tests and Mann-Whitney U tests with post hoc Bonferroni correction were used to compare CIPN between patients according to blood-based factor normative ranges. Linear regression was used to identify blood-based and clinical associations with CIPN development.

**Results:**

The study included 333 participants (266 [79.9%] women; median [interquartile range] age, 58 [18] years) who were consecutively recruited and referred (228 treated with paclitaxel, 105 treated with oxaliplatin; 138 [41.4%] with breast cancer, 83 [24.9%] with colorectal cancer). Most participants had grade 1 CIPN or higher (238 [71.5%] participants). Participants with low hemoglobin pretreatment had worse CIPN posttreatment (median [IQR] composite neurological grading scale score, 5 [2-8] vs 4 [1-6]; *P* = .002; grooved pegboard mean [SD] time, 84.2 [28.7] vs 72.9 [21.1] seconds; *P* = .002; grating orientation task, 4.8 [2.8] vs 3.9 [1.8] mm; *P* = .03; 2-point discrimination, 45% vs 28%; *P* = .01), with no other impairments outside normative ranges associated with CIPN. In the multivariable model, several factors were associated with worse CIPN (*F*_4,315_ = 18.6; *P* < .001; *r^2^* = .19) including for lower hemoglobin (β = −0.47; 95% CI, −0.73 to −0.21; *P* < .001), higher body mass index (β = 0.08; 95% CI, 0.02 to 0.12; *P* = .007), older age (β = 0.08; 95% CI, 0.06 to 0.11; *P* < .001), and female sex (β = −1.08; 95% CI, −1.76 to −0.16; *P* = .01).

**Conclusions and Relevance:**

The results of this cohort study suggest that participants with low pretreatment hemoglobin, higher body mass index, older age, and female sex were more likely to develop paclitaxel- or oxaliplatin-induced CIPN posttreatment. Future research should investigate prospectively whether these risk factors are associated with a higher incidence of CIPN development.

## Key Points

**Question:**

Are pretreatment blood-based and clinical factors associated with chemotherapy-induced peripheral neuropathy (CIPN) persistence posttreatment in patients who have received paclitaxel or oxaliplatin?

**Findings:**

In this cohort study of 333 participants, a greater severity of CIPN was associated with participants who displayed lower pretreatment hemoglobin and higher body mass index, as well as with older patients and among women.

**Meaning:**

The findings of this study suggest that patients with low hemoglobin, higher body mass index, older age, or female sex receiving paclitaxel or oxaliplatin should receive closer monitoring in an attempt to mitigate the development of long-term CIPN.

## Teaser

This cohort study of patients with cancer treated with paclitaxel or oxaliplatin assesses blood-based and clinical factors associated with chemotherapy-induced peripheral neuropathy persistence posttreatment.

## Introduction

The survival rate of cancer continues to rise, with adult cancer survival around 70%.^1^ Accordingly, there are a growing population of cancer survivors who experience acute and chronic toxic effects from curative treatment. Chemotherapy-induced peripheral neuropathy (CIPN) is a dose-limiting and common adverse effect from numerous chemotherapies, including taxanes and platinum agents.^2,3^ CIPN symptoms include sensory, motor, or autonomic effects and commonly present in the hands and feet. Severe CIPN results in dose reductions and treatment terminations, potentially affecting survival.^2,4,5^ Neuropathy can be long lasting and may worsen after treatment,^6,7^ leading to disability or an impact on activities of daily living that diminish quality of life.^8,9^ In order to reduce the incidence of long-term CIPN,^10,11^ there is a need to identify individual risk factors.

Numerous studies have examined potential CIPN risk factors, including genetic variations^12,13^ and clinical and demographic factors.^14,15^ However, there remains no consensus on the full profile of risk factors. Potential blood-based associations with CIPN include serum micronutrients vitamin E, vitamin D, and prealbumin,^16^ suggesting nutritional status may mediate aspects of long-term neurological recovery. In addition, pretreatment anemia^14^ and altered neutrophil-to-lymphocyte ratios^17^ have been reported as prognostic factors of severe CIPN. Other factors may present a risk for developing CIPN including the presence of metabolic conditions (ie, type 2 diabetes^15^ and obesity^18,19^) and older age.^15,20^

Identifying prognostic factors for increased risk of developing CIPN would allow clinicians to monitor at-risk patients more closely and personalize treatment according to risk of toxic effects. In particular, identifying factors associated with long-term, persistent CIPN posttreatment may allow intervention opportunities that promote quality of life in cancer survivorship. The objective of this study was to investigate the association of pretreatment blood-based and clinical factors with CIPN persistence posttreatment in patients who have received paclitaxel or oxaliplatin. We hypothesized that a combined analysis that included a suite of baseline clinical and blood-based factors would identify the most relevant factors associated with risk of developing CIPN, evaluated using the Total Neuropathy Score-clinical (TNSc).

## Methods

### Participants

Participants aged 18 years or older with stage I to IV disease who completed paclitaxel or oxaliplatin chemotherapy 3 to 12 months prior were eligible from Sydney, Australia, or Brisbane, Australia, hospitals between September 2015 and February 2020. The study was approved by South Eastern Sydney Local Health District and Sydney Local Health District (RPAH zone) human research ethics committees, with written informed consent obtained from each participant. This study followed the Strengthening the Reporting of Observational Studies in Epidemiology (STROBE) reporting guideline.

### Neuropathy Assessment

All participants underwent a single comprehensive neurological examination (ie, neurophysiological, clinical-graded, functional, and patient-reported outcomes) 3 to 12 months after chemotherapy completion. CIPN was objectively assessed using the TNSc (Johns Hopkins University), a composite tool of 6 domains including upper and lower limb pin-prick sensory and vibration sensibility, deep tendon reflexes, strength assessment, and patient-reported symptoms.^21,22^ Each domain is graded between 0 (normal) and 4 (severe), combining for a total score between 0 (no symptoms) to 24 (severe symptoms). Researchers completed central training to ensure reliability across assessors. Sensory sural and motor tibial nerve conduction studies were undertaken as in previous research.^23^

Fine motor skills were assessed using the grooved pegboard test to assess time taken for participants to place 25 pegs into grooved holes using the dominant hand, with average time calculated from 2 attempts.^24^ Sensory function was quantified using psychophysical tasks. The grating orientation task used JVP Domes (Stoelting Co). Dome gratings between 0.75 mm and 12 mm were placed onto the index finger either proximal-distally or lateral-medially in random order to identify the smallest grating that could be reliably discriminated. Participants progressed by correctly differentiating between the directions 15 or more times out of 20 attempts.^25^ For 2-point discrimination, an aesthesiometer was placed on the first toe. Participants were required to correctly differentiate between 1 and 2 points (between 2-15 mm) 7 out of 10 times.^26^ Scoring was dichotomized into passing (correctly differentiating ≤15 mm) or failing (not differentiating any level).

Participants self-reported CIPN using the Functional Assessment of Cancer Therapy/Gynecologic Oncology Group-Neurotoxicity questionnaire (FACT/GOG-Ntx-13), a 13-item validated questionnaire rating items on a 5-point Likert scale from “not at all” to “very much” (for a total scale of 52), with lower scores indicating more severe CIPN.^27^

Researchers used the National Cancer Institute Common Terminology Criteria for Adverse Events (CTCAE) neuropathy sensory subscale version 4.0 to grade CIPN severity, including grade 0 (no symptoms), 1 (asymptomatic, not interfering with daily function), 2 (moderate symptoms, limiting daily function), 3 (severe symptoms, limiting daily function and self-care), and 4 (disabling)^28^ at the time of the study assessment.

### Clinical and Blood-Based Risk Factors

Clinical information (ie, age, body mass index [BMI; calculated as weight in kilograms divided by height in meters squared] prior to commencing treatment, cancer type, chemotherapy dose) was retrieved from medical records. Relative dose intensity was calculated by dividing total chemotherapy dose administered by total dose planned as a percentage.^29^ Blood-based results were retrospectively collected from the medical records. The blood test corresponding to the closest date of commencing treatment was selected for analysis (ie, ≤30 days from commencing treatment). Normative reference ranges^30^ included white blood cell count (4.0-10.0 × 10^3^/μL [to convert to × 10^9^/L, multiply by 0.001]), neutrophils (2.0-7.0 × 10^3^/μL), lymphocytes (1.0-3.0 × 10^3^/μL), monocytes (0.2-1.0 × 10^3^/μL), hemoglobin (men: >13 g/dL; women: >12 g/dL [to convert to grams per liter, multiply by 10.0]), albumin (3.3-4.8 g/dL [to convert to grams per liter, multiply by 10.0]), magnesium (1.7-2.7 mg/dL [to convert to millimoles per liter, multiply by 0.4114]) and mean corpuscular volume (MCV, 80.0-100.0 μm^3^ [to convert to femtoliters, multiply by 1.0]). Subclinical markers of inflammation, neutrophil-to-lymphocyte ratio (NLR), and monocyte-to-lymphocyte ratio (MLR) were calculated by dividing the neutrophils and monocytes by lymphocytes, respectively.

### Statistical Analysis

All analyses were conducted using SPSS Statistics Software version 24 (IBM). Descriptive data were presented as means with standard deviations or medians with interquartile range (IQR). Independent samples *t* tests and Mann-Whitney U tests for nonnormally distributed data were used to compare blood-based, clinical, and CIPN outcomes between chemotherapy types, as well as compare CIPN development in those with pretreatment blood parameters outside normal ranges against those without^30^ when more than 10% of results in the sample were outside the normal ranges. Post hoc Bonferroni correction adjustment for multiple comparisons was applied, modifying the significance level from *P* < .05 to *P* < .006 based on the number of contrasts.^31^ Linear regression was used to identify blood-based and clinical (ie, cancer type, treatment, time since treatment, age, BMI) factors associated with CIPN. TNSc was the dependent variable in the univariate and multivariable analyses to assess CIPN on a continuous linear scale, which was normally distributed. Continuous variables associated with TNSc (*P* < .20) in the univariate analysis (except for sex, which was dichotomized) were included in the multivariable model analysis (eTable 1 in the Supplement), with multiple imputation used for missing data. We used backward linear regression to eliminate factors not contributing to the final model (*P* > .10). Variables with *P* < .05 in the multivariable model were considered significant. Normality and data variance were checked using Q-Q residual plots. We defined blood-based outliers using the median absolute deviation, with values within 5 from the median not considered in the multivariate analysis,^32^ resulting in 8 participants excluded based on white blood cell (WBC) counts (28.0, 24.0, 16.0, and 12.3 × 10^3^/μL), lymphocytes (19.0 × 10^3^/μL), neutrophils (26.8 and 11.0 × 10^3^/μL), monocytes (2.8 × 10^3^/μL), NLR (31.5, 26.3, 21.0), and MLR (2.4, 1.4, 1.3). We used the hold-out cross-validation method to evaluate the accuracy of the calculated algorithm when one-third of cases were randomly removed and compared against the other two-thirds.^33^

## Results

### Baseline Demographic Characteristics and Clinical History

A total of 333 participants were included in the study. The recruitment rate from all eligible patients at the main study site was 62.3% (152 of 244 patients), with recruited patients younger than all eligible patients (median [IQR] age, 58 [48-66] vs 64 [53-72] years; *P* < .001) but not differing in gender distribution (78% vs 83%; *P* = .18). Eighty percent of participants were women (266 patients), and the median (IQR) age was 58 (48-66; range, 28-85) years (Table 1). Median treatment duration was 13 (8; range, 2-57) weeks. The most common cancer types were breast (138 [41.4%] patients), colorectal (83 [24.9%]), and ovarian (37 [11.1%]); 17 (5%) patients received prior neurotoxic chemotherapy and 59 (26%) patients who received paclitaxel received carboplatin concurrently.

**Table 1.** Clinical and Demographic Characteristics of Patients Who Received Paclitaxel and Oxaliplatin Chemotherapy

Characteristics	Participants, No. (%)		*P* value
	Paclitaxel (n = 228)	Oxaliplatin (n = 105)	
Women	221 (96.9)	45 (42.9)	<.001
Cancer types			
Breast	138 (60.5)	0	<.001
Colorectal	0	83 (79.0)	
Ovarian	37 (16.2)	0	
Endometrial	32 (14.0)	0	
Gastrointestinal	6 (2.6)	10 (9.5)	
Pancreatic	2 (0.9)	10 (9.5)	
Cervical	3 (1.3)	0	
Lymphoma	0	1 (1.0)	
Unknown	10 (4.4)	1 (1.0)	
Stage			<.001
I	24 (10.5)	1 (1.0)	
II	82 (36.0)	11 (10.5)	
III	70 (30.7)	47 (44.8)	
IV	32 (14.0)	40 (38.1)	
Undefined	20 (8.8)	6 (5.7)	
Type 2 diabetes	18 (7.9)	10 (9.5)	.87
Carpal tunnel syndrome	14 (6.1)	6 (5.7)	.68
Numbness and tingling before chemotherapy	11 (4.8)	2 (1.9)	.007
Blood parameters outside normal range			
Low hemoglobin	47 (20.6)	32 (30.5)	.05
High lymphocytes	27 (11.8)	10 (9.5)	.59
High white blood cell count	20 (8.8)	15 (14.3)	.16
Low lymphocytes	22 (9.6)	8 (7.6)	.85
High neutrophils	11 (4.8)	9 (8.6)	.23
Microcytic anemia	7 (3.1)	11 (10.5)	.02
Low neutrophils	13 (5.7)	1 (1.0)	.006
Low white blood cell count	13 (5.7)	0	.006
Low albumin	9 (3.9)	5 (4.8)	.72
Age, median (IQR), y	57 (48-67)	60 (50-68)	.12
Months since treatment, median (IQR)	9 (6-12)	6 (4-11)	<.001
Chemotherapy dose, mean (SD), mg/m^2^	844.6 (232.4)	749.1 (189.0)	NA
Relative dose intensity, mean (SD), %	88.6 (12.8)	83.8 (13.4)	.03
Body surface area, mean (SD), m^2^	1.81 (0.22)	1.92 (0.23)	<.001
Body mass index, mean (SD)^a^	27.5 (6.6)	27.5 (5.5)	.95
White blood cells, mean (SD), × 10^3^/μL	7.20 (2.7)	7.35 (2.0)	.62
Neutrophils, mean (SD), × 10^3^/μL	4.43 (2.2)	4.71 (1.6)	.24
Lymphocytes, mean (SD), × 10^3^/μL^b^	1.96 (0.8)	1.88 (0.9)	.17
Monocytes, mean (SD), × 10^3^/μL^b^	0.52 (0.3)	0.54 (0.3)	.15
Hemoglobin, mean (SD), g/dL	12.9 (1.2)	12.9 (1.5)	.74
Magnesium, mean (SD), mg/dL	2.07 (0.2)	2.02 (0.2)	.18
Albumin, mean (SD), g/dL	4.0 (0.5)	3.9 (0.5)	.06
Mean corpuscular volume, mean (SD), μm^3^^b^	89.4 (6.4)	86.6 (6.1)	<.001
Neutrophil-to-lymphocyte ratio, mean (SD)^b^	2.7 (2.9)	3.1 (2.4)	.005
Monocyte-to-lymphocyte ratio, mean (SD)^b^	0.31 (0.2)	0.34 (0.2)	.01

Abbreviations: IQR, interquartile range; NA, not applicable.

SI conversion factor: To convert white blood cells, neutrophils, lymphocytes, and monocytes to × 10^9^/L, multiply by 0.001; to convert hemoglobin and albumin to g/L, multiply by 10.0; to convert magnesium to mmol/L, multiply by 0.4114; and to convert mean corpuscular volume to fL, multiply by 1.0.

^a^ Body mass index is calculated as weight in kilograms divided by height in meters squared.

^b^ Mann-Whitney U test for nonnormally distributed data.

### Neurophysiological, Functional, Sensory, and Patient-Reported Neurological Outcomes

Objective and patient-reported neurological assessment outcomes are presented in Table 2. A total of 238 (72.8%) patients reported neuropathy symptoms at median (IQR) 8.0 (7; range, 3-12) months since treatment completion. Overall, 89 participants (27.2%) experienced grade 0 on the CTCAE scale, 117 (35.8%) experienced grade 1, 109 (33.3%) experienced grade 2, and 12 (3.7%) experienced grade 3. Differences in clinical characteristics between those with and without CIPN are presented in eTable 2 in the Supplement. TNSc, CTCAE, FACT/GOG-Ntx-13, grating orientation task, 2-point discrimination, and grooved pegboard scores displayed moderate to strong correlations between all CIPN assessment measures (*r* = 0.31-0.73; all *P* < .001; eFigure in the Supplement).

**Table 2.** Neurological, Sensory, and Functional Outcomes in Patients Who Received Paclitaxel or Oxaliplatin Chemotherapy

Measurement	Total cohort, mean (SD) (N = 333)
TNSc, median (IQR)^a^	4 (2-6)
Sural amplitude, μV^b^	10.8 (8.5)
Tibial amplitude, mV	10.2 (5.0)
Pegboard time, s^b^	76.4 (25.1)
GOT threshold, mm^b^	4.12 (2.1)
FACT/GOG-Ntx-13 score^c^	41.7 (9.3)
CTCAE, No. (%)	
Grade 0	89 (26.7)
Grade 1	117 (35.1)
Grade 2	109 (32.7)
Grade 3	12 (3.6)
Two-point discrimination, fail, No. (%)	93 (33)^d^

Abbreviations: CTCAE, Common Terminology Criteria for Adverse Events; FACT/GOG-Ntx-13, Functional Assessment of Cancer Therapy-Neurotoxicity 13 questionnaire; GOT, grating orientation task; TNSc, Total Neuropathy Score-clinical.

^a^ TNSc is evaluated on a scale from 0 to 24.

^b^ Mann-Whitney U test for nonnormally distributed data.

^c^ The FACT/GOG-Ntx-13 questionnaire is evaluated on a scale from 0 to 52.

^d^ This percentage is based on a subsample of patients.

### Blood-Based Parameters Outside Normative Ranges

Before commencing chemotherapy, some participants demonstrated blood parameters outside normative ranges, including 81 (24.5%) participants with reduced hemoglobin, 36 (10.8%) participants with high lymphocyte counts, 35 (10.5%) participants with high white blood cell counts, and 30 (9.0%) participants with low lymphocyte counts (Table 1). There were no differences in CIPN severity between patients with pretreatment lymphocyte counts or white blood cell counts outside normative reference ranges compared with the remaining cohort. Compared with patients with hemoglobin levels in the reference range, those with reduced pretreatment hemoglobin demonstrated significantly higher TNSc scores (median [IQR] score, 5 [2-8] vs 4 [1-6]; *P* = .002), longer grooved pegboard times (mean [SD], 84.2 [28.7] vs 72.9 [21.1] seconds; *P* = .002), lower grating orientation task results (4.8 [2.8] vs 3.9 [1.8] mm; *P* = .03), and higher failure rates on 2-point discrimination (45% vs 28%; *P* = .01) (Table 3), indicating higher neuropathy burden among the group with reduced hemoglobin.

**Table 3.** Comparisons in CIPN Development by Pretreatment Blood-Based Impairment Status Among Patients Treated With Paclitaxel and Oxaliplatin

Measurement	Reduced hemoglobin			Low lymphocyte count			High lymphocyte count			High white blood cell count		
	Yes (n = 81)	No (n = 252)	*P* value	Yes (n = 33)	No (n = 300)	*P* value	Yes (n = 36)	No (n = 297)	*P* value	Yes (n = 35)	No (n = 298)	*P* value
TNSc, median (IQR)	5 (2-8)	4 (1-6)	.002^a^^,^^b^	5 (2-7)	4 (2-6)	.63	3 (0-6)	4 (2-6)	.11	3 (1-5)	4 (2-6)	.09
Sural amplitude, mean (SD), μV^c^	9.7 (8.8)	11.3 (8.4)	.05^a^	9.5 (8.2)	11.0 (8.6)	.33	10.5 (7.2)	10.9 (8.7)	.97	9.7 (6.1)	10.9 (8.7)	.78
Tibial amplitude, mean (SD), mV	9.8 (4.9)	10.5 (5.0)	.36	9.6 (5.0)	10.4 (5.0)	.45	9.7 (6.0)	10.4 (4.8)	.54	9.9 (5.0)	10.3 (5.0)	.71
CTCAE grade [0-4], median (IQR)	1 (1-2)	1 (0-2)	.07	1 (1-2)	1 (0-2)	.22	1 (0-2)	1 (0-2)	.72	1 (1-2)	1 (0-2)	.90
Pegboard time, mean (SD), s^c^	84.2 (28.7)	72.9 (21.1)	.002^a^^,^^b^	75.1 (16.9)	76.2 (25.4)	.32	78.5 (21.1)	75.8 (25.1)	.12	74.6 (22.2)	76.6 (25.4)	.87
GOT threshold, mean (SD), mm^c^	4.8 (2.8)	3.9 (1.8)	.07	4.1 (1.8)	4.1 (2.1)	.58	4.5 (1.6)	4.1 (2.2)	.03^a^	4.4 (2.0)	4.1 (2.1)	.11
FACT/GOG-Ntx-13 score, mean (SD)^d^	40.4 (8.4)	42.5 (9.1)	.06	40.0 (8.9)	42.1 (9.0)	.18	41.7 (7.1)	41.9 (9.1)	.86	42.2 (8.3)	41.7 (9.4)	.71
2-point discrimination, fail, %	45	28	.01^a^	43	32	.23	31	33	.86	20	34	.09

Abbreviations: CIPN, chemotherapy-induced peripheral neuropathy; CTCAE, Common Terminology Criteria for Adverse Events; FACT/GOG-Ntx-13, Functional Assessment of Cancer Therapy-Neurotoxicity 13 questionnaire; GOT, grating orientation task; TNSc, Total Neuropathy Score-clinical.

^a^ *P* ≤ .05.

^b^ Significant after applying Bonferroni correction.

^c^ Mann-Whitney U test for nonnormally distributed data.

^d^ The FACT/GOG-Ntx-13 questionnaire is evaluated on a scale from 0 to 52.

### Factors Associated With CIPN Using Total Neuropathy Score

**Figure 1.**
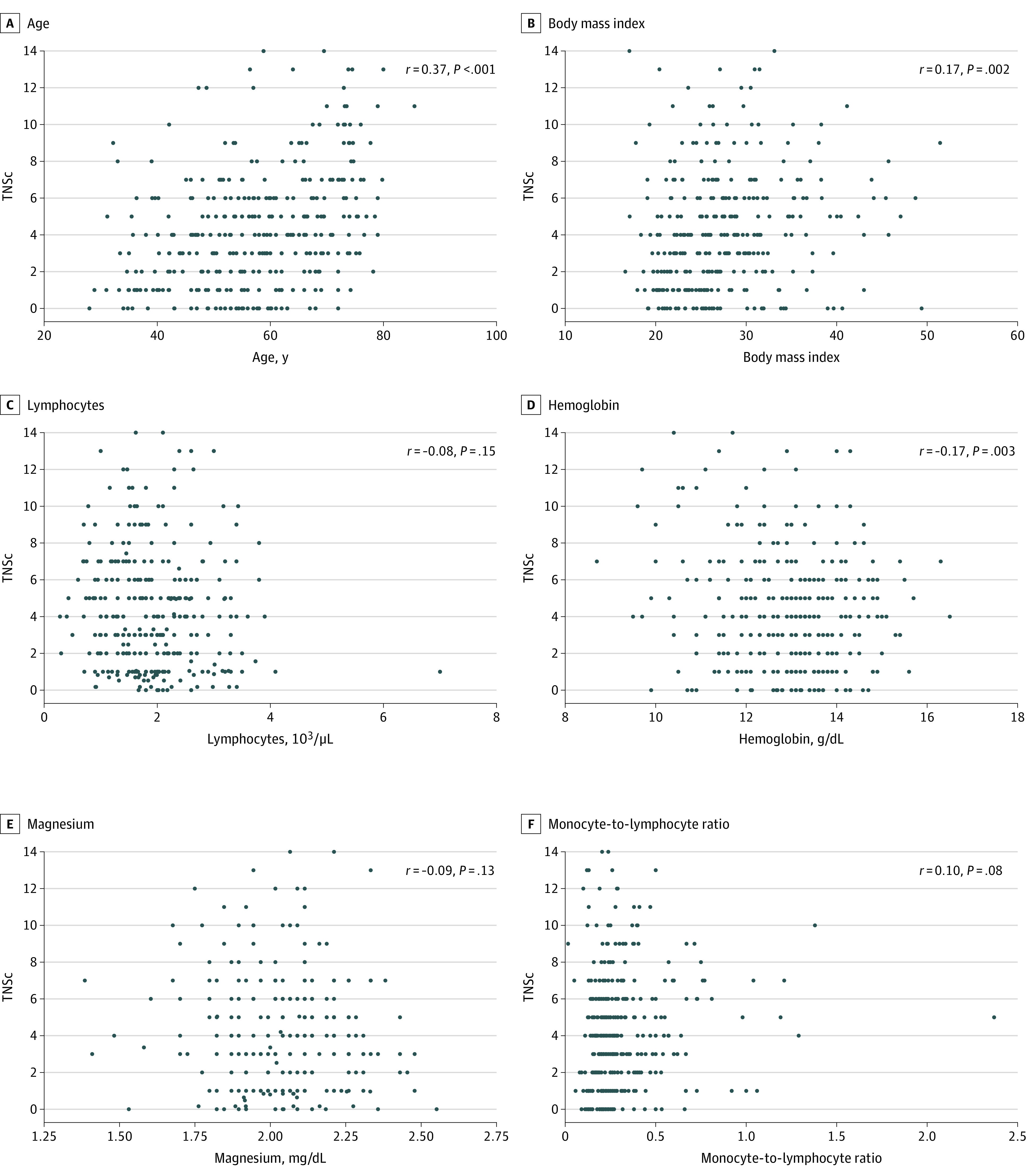
Blood-Based and Clinical Factor Univariate Correlates of Total Neuropathy Score-clinical (TNSc) Across the Whole Cohort To convert lymphocytes to × 10^9^/L, multiply by 0.001; hemoglobin to grams per liter, multiply by 10.0; and magnesium to millimoles per liter, multiply by 0.4114.

**Figure 2.**
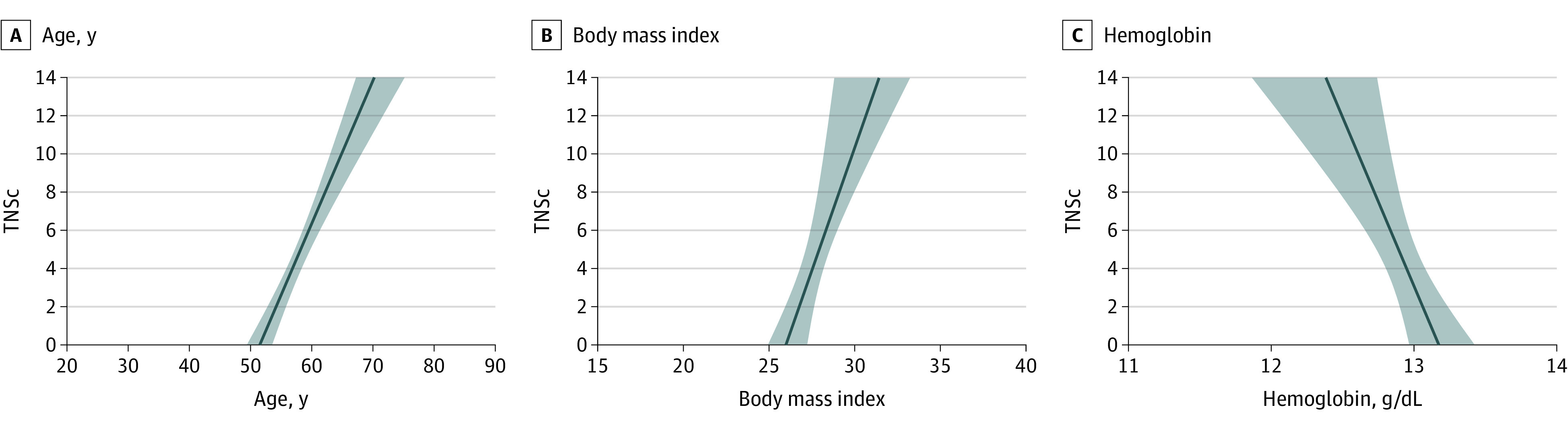
Projected Values of Mean TNSc Scores Using a Fitted Model of Blood-Based and Clinical Factors From the Multivariable Model The association of each factor with Total Neuropathy Score-clinical (TNSc) is influenced by the other explanatory variables in the multivariable model. TNSc scores range from 0 (no CIPN symptoms) to 24 (severe CIPN symptoms).

Univariate blood-based and clinical CIPN correlates are presented in Figure 1. A multivariable model found a significant association of baseline blood-based and clinical factors with CIPN, measured using TNSc (*F*_4,315_ = 18.6; *P* < .001; *r^2^* = 0.19) (eTable 3 in the Supplement). Factors associated with higher TNSc (worse CIPN) were older age (β = 0.08; 95% CI, 0.06 to 0.11; *P* < .001), higher BMI (β = 0.08; 95% CI, 0.02 to 0.12; *P* = .007), lower hemoglobin (β = −0.47; 95% CI, −0.73 to −0.21; *P* < .001), and female sex (β = −1.08; 95% CI, −1.76 to −0.16; *P* = .01) (Figure 2). Nonsignificant factors included dose, relative dose intensity, months posttreatment, WBC, neutrophils, lymphocytes, monocytes, albumin, magnesium, MCV, NLR, and MLR.

### Factors Associated With CIPN by Chemotherapy Type

To determine if these associations were the same in both paclitaxel (228 participants) and oxaliplatin (105 participants) treated cohorts, subgroup analyses were conducted. In total, 66% of paclitaxel and 88% of oxaliplatin-treated patients reported neuropathy symptoms. Paclitaxel- and oxaliplatin-treated participants differed in sex, cancer type, disease stage, time since treatment completion, relative dose intensity, BSA, pretreatment MCV, MLR, and NLR. There were no differences in pretreatment hemoglobin, BMI, and age between groups.

In the paclitaxel-treated group, the multivariable model found an association with CIPN (*F*_3,216_ = 18.8; *P* < .001; *r^2^* = 0.21). Factors associated with higher TNSc were older age (β = 0.09; 95% CI, 0.06 to 0.12; *P* < .001), higher BMI (β = 0.08; 95% CI, 0.03 to 0.14; *P* = .004), and lower hemoglobin (β = −0.39; 95% CI, −0.71 to −0.07; *P* = .013).

In the oxaliplatin-treated group, the multivariable model found an association with CIPN (*F*_3,96_ = 6.45; *P* < .001; *r^2^* = 0.17). Factors associated with higher TNSc were older age (β = 0.09; 95% CI, 0.04 to 0.14; *P* = .001), lower hemoglobin (β = −0.60; 95% CI, −1.04 to −0.16; *P* = .007), and higher albumin (β = 1.89; 95% CI, 0.44 to 3.33; *P* = .009).

## Discussion

This study investigated pretreatment blood-based and clinical risk factors for developing CIPN following paclitaxel or oxaliplatin treatment. In our cohort, CIPN symptoms were highly prevalent and comparable with similarly treated populations.^16^ The factors most consistently associated with severe CIPN were low hemoglobin, higher BMI, and older age. However, different factors associated with CIPN were also identified in chemotherapy-specific analyses, highlighting the potential differences in mechanisms underlying CIPN development with different agents. While no individual marker stood out as a strong prognostic factor for CIPN, it is likely that multiple markers contribute as prognostic factors for CIPN risk.

In both paclitaxel- and oxaliplatin-treated patients, low hemoglobin associated with more severe CIPN. Hemoglobin below normative ranges was identified in 24.5% of patients, and CIPN was more severe in this group, measured using clinical (TNSc) and functional (grooved pegboard) assessments. Oxaliplatin-induced CIPN severity has previously been associated with low baseline hemoglobin.^14,34,35,36^ Similarly, anemia has been identified as a risk factor for vincristine-induced CIPN.^37^ Severe CIPN incidence was also greater in paclitaxel-treated patients with treatment-emergent anemia.^38^ However, no prior studies have investigated the association of baseline hemoglobin with CIPN in paclitaxel-treated patients. Interestingly, low hemoglobin has also been associated with diabetic neuropathy,^39^ suggesting that hemoglobin may be linked to neuropathy across different etiologies.

It is difficult to identify the most plausible pathophysiological explanation for the association of low hemoglobin with CIPN development. Cancer-associated anemia is complex and associated with nutritional iron deficiency but also chronic disease.^40^ It is plausible that the association between CIPN and hemoglobin in our cohort is linked to other factors. Pretreatment anemia could be a surrogate for other nutritional deficiencies, comorbidities, or general health, with anemic patients with breast cancer shown to have worse clinical cancer outcomes than nonanemic patients.^41^ Neuropathy is also associated with iron-deficiency anemia, as well as with improved neuropathic symptoms and nerve conduction parameters occurring with iron therapy.^42,43^ However, serum iron or ferritin levels were not available in our cohort. Of note, MCV was not reduced in our cohort, suggesting widespread microcytic anemia and iron deficiency were unlikely.^44^ Importantly, future studies examining the relationship between iron levels, anemia, and CIPN are needed in prospective cancer cohorts exposed to neurotoxic chemotherapies.

Higher albumin was associated with higher CIPN severity among oxaliplatin-treated patients, although the association was weak. Although no previous CIPN studies displayed this relationship, patients with diabetes and with high albumin have been shown to be at higher risk for developing diabetic-neuropathy, likely due to oxidative stress and inflammation.^45^ Conversely, previous studies of oxaliplatin-treated patients found lower albumin associated with higher CIPN severity,^14,34,35^ likely representing impaired nutritional status and inadequate protein intake. Of note, only 4% of participants presented with hypoalbuminemia in our study compared with 80% in the Vincenzi study, and with lower levels of diabetes and anemia, suggesting considerable differences in nutritional status and morbidity between cohorts.^34^ As albumin fluctuates postsurgery or with morbidity, it is difficult to determine its impact on CIPN in absence of other concomitant factors. Accordingly, these cross-sectional findings should be verified through prospective evaluation.

Like previous studies, we found an association between increasing age and CIPN severity in paclitaxel- and oxaliplatin-treated patients. Increased risk for developing neuropathy in older patients has been identified in large-scale studies of oxaliplatin-treated^20^ and paclitaxel-treated patients,^15,46,47^ with 4% increased odds of developing CIPN with each increasing year in paclitaxel-treated patients.^15^ However, not all studies found this association. No association between age and oxaliplatin-induced CIPN^48^ and longer CIPN duration has been identified in younger colorectal cancer patients.^34^ Another large-scale study of paclitaxel-treated patients did not identify age as an independent CIPN risk factor,^18^ although only 12% of patients were aged 65 years or older.

Severe CIPN associated with higher BMI in our cohort and the paclitaxel-specific analysis, similar to previous research.^18,49^ We did not find a significant association between BMI and CIPN in oxaliplatin-treated patients. Baseline BMI has been associated with oxaliplatin-induced CIPN in some studies,^50^ but not other oxaliplatin cohort studies.^51,52^ However, often other obesity measures including higher body surface area (≥2.0 m^2^) were linked to oxaliplatin-induced CIPN.^19,51,53^ Obesity is associated with increased idiopathic neuropathy risk^54^ and metabolic dysregulation, hyperinsulinemia, and insulin sensitivity, which can also predispose patients to neuropathy,^55^ so there is a mechanistic rationale for increased CIPN risk. Additionally, the links between obesity and CIPN may be partially mediated via higher treatment doses administered to patients with higher body surface area.^49^ While we did not find dose or relative dose intensity were associated with developing CIPN, cumulative dose is generally associated with greater CIPN risk.^4^ Within specific regimens, small cumulative dose variations may be less often associated with CIPN severity, potentially reflecting the presence of multiple risk factors.

In addition to blood and clinical factors discussed above, we also identified that WBC, neutrophils, lymphocytes, monocytes, magnesium, MCV, NLR, and MLR were not associated with CIPN across either chemotherapy type. In future studies, analyses of other blood parameters associated with hemoglobin including red blood cells, hematocrit, or iron levels may assist in determining clearer links between hemoglobin and CIPN. Further, we did not find associations between total dose, relative dose intensity, or time posttreatment with CIPN severity. While dose and timing factors have been shown to be relevant to CIPN incidence,^3^ the lack of association in this study and previous studies^56,57^ likely highlights the complexity of CIPN development. It is likely that a suite of interconnected risk factors are associated with CIPN development and that the level of predisposing risk varies across individuals. Further, genetic factors may provide additional independent prognostic value of CIPN risk.^12,13^ Although numerous single nucleotide variations (SNVs) associated with CIPN have been identified across neurotoxic chemotherapies, effect sizes are often small and further validation studies are needed.^13^ It is likely that multiple genes and SNVs, rather than a single SNV, will contribute to prognostic CIPN risk models,^13^ similar to the present findings of multiple clinical factors contributing to CIPN risk.

### Strengths and Limitations

This was a multisite, large-scale study, incorporating comprehensive CIPN assessment using numerous validated objective and patient-reported assessment tools including relevant neurophysiologic measures.^56,58^ Factors identified in our model using backward regression were validated using the hold-out method and consistent when using forward regression, providing reassurance in the factors identified.

This study still had several limitations. Our conclusions are limited to the 2 chemotherapy drugs commonly associated with CIPN and may not be applicable to other CIPN-inducing therapies. Because of the large sample of paclitaxel-treated breast and gynecological cancer and oxaliplatin-treated patients with colorectal cancer, our findings are predominantly generalized to women and these cancer types, which may limit the generalizability more broadly. We did not collect data on nutritional supplementation, transfusions, or other interventions that may potentially affect CIPN development. We acknowledge the inherent limitations of cross-sectional CIPN assessments, and these findings should be confirmed in prospectively assessed cohorts.

## Conclusions

Our findings are clinically relevant, encouraging consideration of age and body composition in addition to anemia when prescribing potentially neurotoxic chemotherapy regimens. Irrespective of causation, CIPN results in substantial long-term morbidity, consistently rated as a key contributor to adverse outcomes in long-term survivors.^59^ Closer monitoring of those at higher risk in order to allow dose modification may mitigate the development of long-term CIPN among patients receiving paclitaxel or oxaliplatin. Enhanced identification of patients at risk of long-term neurotoxicity is critical to enable the development of personalized treatment approaches. Importantly, the risk factors identified in our analysis are routinely available in clinical practice without additional specialized assessments. Prospective validation of these risk factors to examine the benefit of closer neurological surveillance of those with substantial CIPN risk is an important next step. Future examination of the role of intervention to correct prospectively validated risk factors (ie, hemoglobin level) may be warranted in the future to investigate the impact on long-term CIPN outcomes in cancer survivors.

## Notes

Supplement. **eTable 1.** Neurological, Sensory, and Functional Outcomes, Including Comparisons Between Patients Who Received Paclitaxel and Oxaliplatin Chemotherapy **eTable 2.** Clinical and Demographic Characteristics Comparison Between Patients With TNSc<4 Versus Those With TNSc≥4 **eTable 3.** Linear Regression to Determine Univariate Factors Influencing the Development of Chemotherapy Induced Peripheral Neuropathy Contributing to the Multivariable Model **eFigure.** Correlation Matrix Between Chemotherapy-Induced Peripheral Neuropathy Outcome Measures
