# Supplementary material for: Hemoglobin, Body Mass Index, and Age as Risk Factors for Paclitaxel- and Oxaliplatin-Induced Peripheral Neuropathy
Source: JAMA Netw Open. 2021 Feb 15;4(2):e2036695. doi: 10.1001/jamanetworkopen.2020.36695 (PMC7885037; doi:10.1001/jamanetworkopen.2020.36695)
Supplement: Supplement. — eTable 1. Neurological, Sensory, and Functional Outcomes, Including Comparisons Between Patients Who Received Paclitaxel and Oxaliplatin Chemotherapy eTable 2. Clinical and Demographic Characteristics Comparison Between Patients With TNSc<4 Versus Those With TNSc≥4 eTable 3. Linear Regression to Determine Univariate Factors Influencing the Development of Chemotherapy Induced Peripheral Neuropathy Contributing to the Multivariable Model eFigure. Correlation Matrix Between Chemotherapy-Induced Peripheral Neuropathy Outcome Measures [file jamanetwopen-e2036695-s001.pdf]

Supplemental Online Content

Mizrahi D, Park SB, Li T, et al. Hemoglobin, body mass index, and age as risk factors for paclitaxel- and oxaliplatin-induced peripheral neuropathy. *JAMA Netw Open*. 2021;4(2):e2036695. doi:10.1001/jamanetworkopen.2020.36695

eTable 1. Neurological, Sensory, and Functional Outcomes, Including Comparisons Between Patients Who Received Paclitaxel and Oxaliplatin Chemotherapy

eTable 2. Clinical and Demographic Characteristics Comparison Between Patients With TNSc<4 Versus Those With TNSc≥4

eTable 3. Linear Regression to Determine Univariate Factors Influencing the Development of Chemotherapy Induced Peripheral Neuropathy Contributing to the Multivariable Model

eFigure. Correlation Matrix Between Chemotherapy-Induced Peripheral Neuropathy Outcome Measures

This supplemental material has been provided by the authors to give readers additional information about their work.

© 2021 Mizrahi D et al. *JAMA Network Open.*

eTable 1. Neurological, sensory, and functional outcomes, including comparisons between patients who received paclitaxel and oxaliplatin chemotherapy

	**Paclitaxel (n=228)**		**Oxaliplatin (n=105)**		**p-value**
	**Mean**	**SD**	**Mean**	**SD**	
TNSc (0-24, median/IQR)	4	5	5	4	**.005**$^{*+}$
Sural amplitude (µV)^	13.2	8.9	5.7	4.5	**<.001**$^{*+}$
Tibial amplitude (mV)	10.6	5.1	9.4	4.5	**.05***
Pegboard time (s)^	71.4	20.5	87.8	30.4	**<.001**$^{*+}$
GOT threshold (mm)^	3.6	1.5	5.3	2.6	**<.001**$^{*+}$
FACT/GOG-Ntx-13 (0-52)	42.8	9.3	39.5	8.9	**.003**$^{*+}$
	**n**	**%**	**n**	**%**	
CTCAE<br>Grade 0	76	34	13	12	**<.001**$^{*+}$
Grade 1	73	32	44	43	
Grade 2	69	31	40	39	
Grade 3	6	3	6	56	
Two-point discrimination (fail)	58	29	35	42	**.04***

TNSc = Total Neuropathy Score-clinical, CTCAE = Common Terminology Criteria for Adverse Events, GOT = grating orientation task, FACT = Functional Assessment of Cancer Therapy, WBC = white blood cell. * = p≤.05. $^{+}$ = significant after applying Bonferroni correction. ^Mann-Whitney U test for not normally distributed data

© 2021 Mizrahi D et al. *JAMA Network Open.*

eTable 2. Clinical and demographic characteristics comparison between patients with TNSc<4 versus those with TNSc≥4

	**Patients with TNSc<4 (n=150)**		**Patients with (TNSc≥4) (n=180)**		**p-value**
	**n**	**%**	**n**	**%**	
Sex, Female	128	85	134	76	**.04**
Cancer Types					.31
Breast	75	50	62	34	
Colorectal	28	19	53	29	
Ovarian	16	11	21	12	
Endometrial	14	9	18	10	
Unknown/Other	17	11	26	15	
Chemotherapy<br>Paclitaxel<br>Oxaliplatin	114<br>36	76<br>24	113<br>67	63<br>37	**.009**
Stage					.12
I	8	5	17	9	
II	60	40	33	18	
III	46	31	70	39	
IV	30	20	41	23	
Undefined	6	4	19	11	
Type 2 diabetes	10	7	21	12	.15
Carpal tunnel syndrome	5	4	17	10	**.04**
Numbness and tingling before chemotherapy	8	5	6	3	.35
Blood parameters outside normal range					
Low haemoglobin	28	19	51	29	**.04**
High lymphocytes	18	12	18	10	.47
High white blood cell count	18	12	16	9	.36
Low lymphocytes	13	9	20	11	.44
High neutrophils	7	5	12	7	.61
Microcytic anaemia	7	5	11	6	.91
Low neutrophils	6	4	9	5	.66
Low white blood cell count	7	5	7	4	.53
Low albumin	6	4	9	5	.84
	**Mean**	**SD**	**Mean**	**SD**	**p-value**
Age (years, median/IQR)	54	18	62	18	**<.001**
Months since treatment (median/IQR)	8	7	7.5	8	.36
Relative dose intensity (%)	88.5	12.3	86.3	13.7	.27
Dose (mg)<br>Paclitaxel<br>Oxaliplatin	1481<br>1329	418<br>439	1528<br>1438	443<br>371	 .42<br>.20
Body surface area (m^2)	1.80	0.21	1.89	0.23	**<.001**

© 2021 Mizrahi D et al. *JAMA Network Open.*

Body mass index (kg/m^2)	26.0	5.5	28.9	6.6	**<.001**

**3 participants without a complete TNSc assessment were removed from all analyses and not included in this table.*

© 2021 Mizrahi D et al. *JAMA Network Open.*

eTable 3. Linear regression to determine univariate factors influencing the development of chemotherapy induced peripheral neuropathy contributing to the multivariable model

	Whole cohort (n=325)			Paclitaxel (n=221)			Oxaliplatin (n=104)		
	Univariate	Multivariable		Univariate	Multivariable		Univariate	Multivariable	
	p-value	B (95% C.I.)	p-value	p-value	B (95% C.I.)	p-value	p-value	B (95% C.I.)	p-value
Age	**<.001***	0.08 (0.06 to 0.11)	**<.001***	**<.001***	0.09 (0.06 to 0.12)	**<.001***	**.004***	0.09 (0.04 to 0.14)	**.001***
Sex	**.06***	-1.08 (-1.76 to -0.16)	**.01***	.67			.80		
Body mass index	**.002***	0.08 (0.02 to 0.12)	**.007***	**.001***	0.08 (0.03 to 0.14)	**.004***	.14		
Chemotherapy dose (mg/m^2)	.84			.93			.36		
Relative dose intensity	.51			.64			.91		
Months post treatment	.32			.92			.60		
White blood cells	.28			.82			.15		
Neutrophils	.25			.53			.08		
Lymphocytes	.15			.31			.47		
Monocytes	.67			.99			.53		
Haemoglobin	**.003***	-0.47 (-0.73 to -0.21)	**<.001***	**.008***	-0.39 (-0.71 to -0.07)	**.013***	**.07***	-0.60 (-1.04 to -0.16)	**.007***
Albumin	.35			.59			**.18***	1.89 (0.44 to 3.33)	**.009***
Magnesium	.13			.37			.32		
MCV	.46			.72			.46		
Neutrophil-to-lymphocyte ratio	.83			.89			.53		
Monocyte-to-lymphocyte ratio	.08			.23			.22		

*Factors included in final backwards logistic multivariable model. Univariate factors with p<.2 that had p<.1 were not included in the final model and removed during the backwards removal model process. Negative sex value denotes female gender.

© 2021 Mizrahi D et al. *JAMA Network Open.*

eFigure 1. Correlation matrix between chemotherapy-induced peripheral neuropathy outcome measures

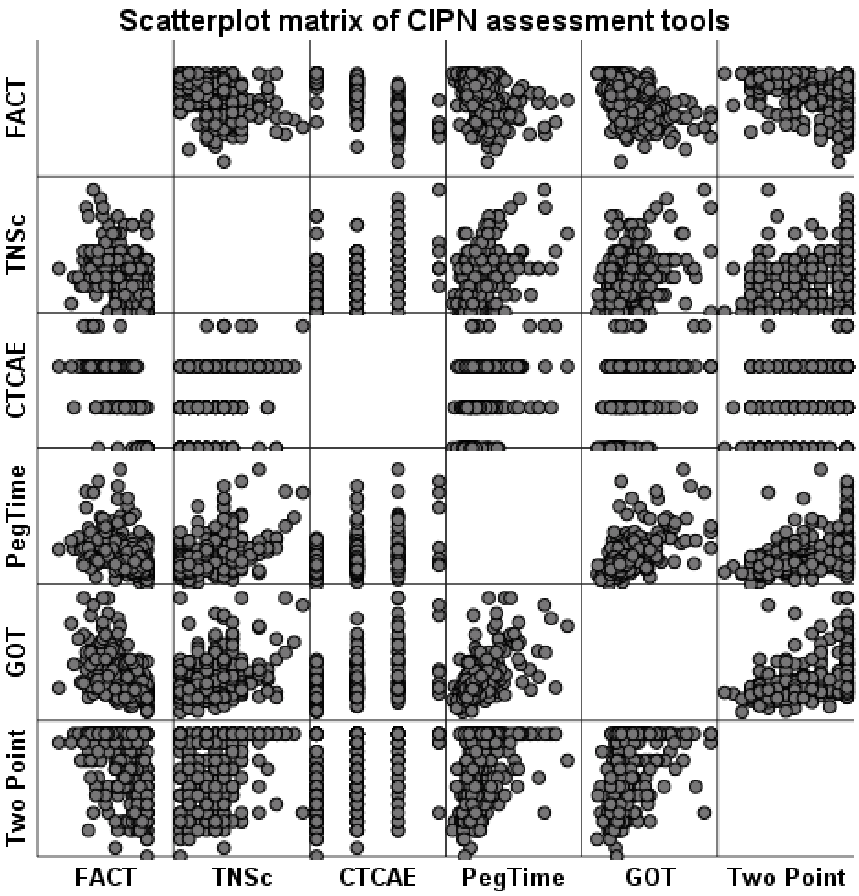

FACT=FACT/GOG-Ntx-13, TNSc=Total Neuropathy Score-clinical, CTCAE=Common Terminology Criteria for Adverse Events, PegTime=Grooved Pegboard, GOT=grating orientation task, Two Point=Two-point discrimination

© 2021 Mizrahi D et al. *JAMA Network Open.*
